# Supplementary material for: Dynalign II: common secondary structure prediction for RNA homologs with domain insertions
Source: Nucleic Acids Res. 2014 Nov 21;42(22):13939–48. doi: 10.1093/nar/gku1172 (PMC4267632; doi:10.1093/nar/gku1172)
Supplement: SUPPLEMENTARY DATA [file supp_gku1172_nar-02021-z-2014-File012.zip › manual/GUI/html/TurboFold.html]

RNAstructure GUI Help -- TurboFold


|  |  |  |
| --- | --- | --- |
|  | RNAstructure GUI Help TurboFold | - Contents - Index |
| TurboFold is a tool that will find the lowest free energy secondary structure common to multiple (two or more) unaligned sequences, using partition function calculations. The input is two or more sequences. The output is an equivalent number of structures (saved as CT files).   ---  **To predict the common structures with TurboFold**   1. Click the button labeled "Sequence File" and provide the name of a sequence file. 2. A default name has been entered for the CT File. If desired, this name can be changed by clicking the corresponding button. 3. Click the "ADD" button to add the sequence and CT files to the sequence set under consideration. Note that at least three sequences must be added for TurboFold to run correctly. 4. Continue adding sequence and CT files to the sequence set in this manner until all desired input and output files have been specified. 5. If a sequence/CT pair was entered in error, it can be removed by clicking the "Delete Sequence" button. The number of the sequence to be removed can be found in the text box next to the button, and it is by default the last sequence. However, specific sequence/CT pairs can be deleted by changing this value. Only one sequence can be deleted at a time. 6. Choose the mode TurboFold should run in. There are three modes, each with its own set of parameters. The modes and their parameters are described below, as well as a group of general parameters. The default mode for TurboFold is Maximum Expected Accuracy. 7. Click "Start" to begin the TurboFold algorithm. This calculation may take some time. A progress bar opens to indicate that the calculation is running. 8. When complete, windows will open to display the structures predicted for each sequence.  ---   **General Parameters for TurboFold**  Gamma  A general optimized constant that balances how much information is used from other sequences when predicting the common structure for a given sequence.  Iterations  The number of iterations the TurboFold calculation should go through in refining the prediction of the common structure.   --- | | |

| Visit The Mathews Lab RNAstructure Page for updates and latest information. | | |
